# The role of atrial fibrillation in vascular cognitive impairment and dementia: epidemiology, pathophysiology, and preventive strategies

**DOI:** 10.1007/s11357-024-01290-1

**Published:** 2024-08-13

**Authors:** Mónika Fekete, Eric M. Liotta, Tihamer Molnar, Gábor A. Fülöp, Andrea Lehoczki

**Affiliations:** 1 Institute of Preventive Medicine and Public Health, Semmelweis University, Budapest, Hungary; 2 Doctoral College, Health Sciences Program, Semmelweis University, Budapest, Hungary; 3 Department of Neurology, Northwestern University, Chicago, IL USA; 4 Department of Anaesthesiology and Intensive Care, Medical School, University of Pecs, Pecs, Hungary; 5 Heart and Vascular Center, Semmelweis University, Budapest, Hungary

**Keywords:** Atrial fibrillation, VCI, Vascular cognitive impairment, Emboli, VCID, Cognitive impairment

## Abstract

The aging population in Europe faces a substantial burden from dementia, with vascular cognitive impairment and dementia (VCID) being a preventable cause. Atrial fibrillation (AF), a common cardiac arrhythmia, increases the risk of VCID through mechanisms such as thromboembolism, cerebral hypoperfusion, and inflammation. This review explores the epidemiology, pathophysiology, and preventive strategies for AF-related VCID. Epidemiological data indicate that AF prevalence rises with age, affecting up to 12% of individuals over 80. Neuroimaging studies reveal chronic brain changes in AF patients, including strokes, lacunar strokes, white matter hyperintensities (WMHs), and cerebral microbleeds (CMHs), while cognitive assessments show impairments in memory, executive function, and attention. The COVID-19 pandemic has exacerbated the underdiagnosis of AF, leading to an increase in undiagnosed strokes and cognitive impairment. Many elderly individuals did not seek medical care due to fear of exposure, resulting in delayed diagnoses. Additionally, reduced family supervision during the pandemic contributed to missed opportunities for early detection of AF and related complications. Emerging evidence suggests that long COVID may also elevate the risk of AF, further complicating the management of this condition. This review underscores the importance of early detection and comprehensive management of AF to mitigate cognitive decline. Preventive measures, including public awareness campaigns, patient education, and the use of smart devices for early detection, are crucial. Anticoagulation therapy, rate and rhythm control, and addressing comorbid conditions are essential therapeutic strategies. Recognizing and addressing the cardiovascular and cognitive impacts of AF, especially in the context of the COVID-19 pandemic, is essential for advancing public health.

## Introduction

Europe is aging at an unprecedented rate, presenting significant challenges for public health systems [1]. Among the various age-related diseases, dementia stands out as a particularly burdensome condition [2–6]. Dementia is broadly categorized into two main types: Alzheimer’s disease (AD) and vascular cognitive impairment and dementia (VCID) [5, 7]. AD is a neurodegenerative disorder characterized by the progressive loss of cognitive functions, including memory, language, and executive functions. AD accounts for half of dementia cases and currently affects millions worldwide, with the incidence sharply increasing with age. Unfortunately, AD remains incurable and unpreventable, posing a significant challenge for healthcare systems.

VCID refers to cognitive impairment and dementia resulting from macrovascular and microvascular pathologies, such as stroke and cerebral small vessel disease (CSVD) [8–11]. VCID is a leading cause of dementia and is preventable, which sets it apart from AD [5, 7]. The incidence of VCID increases significantly with age [5, 7]. In Europe, where nearly one-fifth of the population is aged 65 or older, the prevalence of VCID is rising [2]. Understanding the epidemiological trends of VCID is crucial for developing effective preventive and therapeutic strategies. Given that AD remains incurable, it is critical to focus on preventable causes of dementia, such as VCID. Identifying and managing these preventable factors can significantly reduce the burden of dementia on individuals and healthcare systems [6].

Atrial fibrillation (AF) emerges as a significant preventable cause of VCID [12–14]. AF is a common cardiac arrhythmia characterized by an irregular and often rapid heart rate. This arrhythmia can lead to various complications, including thromboembolism and reduced cerebral perfusion, which are directly linked to the development of dementia [12, 14–19].

The goal of this review is to overview the current evidence on the relationship between atrial fibrillation and vascular contributions to cognitive impairment and dementia. We aim to explore the pathophysiological mechanisms linking AF to VCID, examine clinical evidence supporting this connection, and discuss therapeutic implications and future directions for research and treatment. By understanding and addressing AF as a preventable cause of VCID, we can develop more effective strategies to mitigate cognitive decline and improve the quality of life for the aging population.

## Epidemiology of atrial fibrillation

AF is a major concern for the aging population due to its increasing prevalence with advancing age [15–19]. Approximately 2% of the general population is affected by AF, but this figure rises dramatically to 10–12% among those aged 80 and older [20–27]. The condition is more prevalent in men than in women, although the risk for women increases with age [28–30]. Geographical differences in the prevalence of AF also exist [27, 30], with higher rates observed in North America and Europe compared to Asia and Africa. These variations can be attributed to differences in lifestyle, healthcare access, and genetic factors. Socioeconomic factors further influence the prevalence and management of AF. Differences in health literacy, patient preferences, and healthcare demands likely contribute to socioeconomic variations in health outcomes [31]. In wealthier regions, better access to healthcare and early diagnosis contribute to more effective management of AF. In contrast, lower socioeconomic status is associated with higher rates of undiagnosed and untreated AF, exacerbating the risk of complications such as VCID [32–34].

Underdiagnosis of AF is a significant problem, particularly among the elderly [35]. Many cases of AF remain asymptomatic or present with non-specific symptoms, leading to delayed diagnosis and treatment [35–39]. Studies suggest that a substantial proportion of AF cases go undetected, particularly in older adults who are at higher risk for complications. This underdiagnosis is a critical issue as it prevents timely intervention that could mitigate the risk of stroke and cognitive impairment. Addressing the underdiagnosis of AF requires increased awareness and proactive screening, especially in high-risk populations. Utilizing technologies such as wearable devices and implementing regular ECG screenings in routine check-ups can help detect AF early [40–43]. Increased utiliztion of wearable devices may also help clarify the burden of paroxysmal AF that meets the threshold for benefit from therapeutic interventions such as anticoagulation or ablation therapy, and allow for individualized care of AF [44–46]. By improving the detection and management of AF, we can reduce its impact on cognitive health and overall quality of life for the aging population.

## AF, cognitive decline, and dementia: clinical evidence

Numerous epidemiological studies have established a link between AF and an increased risk of cognitive impairment and dementia [15–19, 47]. These studies suggest that AF independently increases the risk of VCID beyond traditional stroke pathways. The presence of AF has been shown to correlate with a higher incidence of dementia, even in the absence of clinically apparent strokes [15–19]. Studies suggest that older female AF patients have a higher risk of developing dementia compared to their male counterparts [48].

### Neuroimaging findings

Neuroimaging studies have provided valuable insights into the brain changes associated with AF [49–54]. Findings such as white matter hyperintensities (WMH), cerebral microhemorrhages (CMHs; also known as cerebral microbleeds), lacunar infarcts, and cortical atrophy are more prevalent in patients with AF [49–55]. WMHs indicate areas of chronic ischemia, while CMHs are signs of microvascular damage. Cortical atrophy, or the thinning of the brain’s cortex, further illustrates the structural brain damage associated with AF [56, 57]. These neuroimaging findings are indicative of the chronic brain damage that contributes to cognitive decline in individuals with AF.

### Cognitive function assessments

Cognitive function tests in patients with AF often reveal impairments in various domains, including memory, executive function, and attention [56–61]. These impairments highlight the multifaceted impact of AF on cognitive health. For instance, memory tests may show deficits in recalling recent events or learning new information, while executive function tests may reveal difficulties in planning, organizing, and completing tasks [56–61]. Attention assessments might indicate problems with maintaining focus or processing information quickly. These cognitive deficits underscore the need for comprehensive management strategies to address the cognitive aspects of AF. By recognizing and addressing these impairments early, healthcare providers can develop targeted interventions to help mitigate the cognitive decline associated with AF.

## Pathophysiology linking AF and VCID

### Thromboembolism

Atrial fibrillation can lead to the formation of blood clots within the hypomotile recesses of the atria. These small clots may travel to the brain, causing ischemic strokes [33, 62–65], which are causally linked to VCID [66]. Of particular concern are multiple microstrokes, which may not produce immediate clinical symptoms but cumulatively cause significant brain damage over time. The individual microemboli causing these microstrokes are likely to be clinically occult; however, in high-risk patients, microemboli might be detectable by transcranial doppler ultrasound as high-intensity transient signals, and the connection between ultrasound identification of microemboli, microemboli burden, and cognitive decline is an area in need of further investigation [67]. These recurrent microstrokes can disrupt brain networks and lead to rapid cognitive decline. The cumulative effect of these microstrokes can result in substantial brain damage, contributing to the progression of cognitive impairment and dementia.

### Cerebral hypoperfusion

Atrial fibrillation may cause fluctuating or reduced blood flow to the brain [68]. This reduced blood flow can lead to the development of white matter lesions and other types of brain damage associated with VCID [68]. Chronic hypoperfusion affects the brain’s ability to function optimally, leading to gradual cognitive decline. One critical concept in understanding cerebral hypoperfusion is the vulnerability of watershed areas. These are regions of the brain located at the border zones between major arterial territories and are particularly susceptible to ischemic injury. In aging, due to structural and functional alterations in the cerebral microcirculation, such as capillary rarefaction, impaired endothelial vasodilation, and neurovascular impairment, there is a significant area at risk for ischemic injury [69–72]. These changes exacerbate the effects of reduced cerebral blood flow in patients with AF, increasing the risk of cognitive decline.

### Inflammation and endothelial dysfunction

Both AF and VCID involve significant inflammatory processes and endothelial dysfunction [73–76]. Systemic inflammation associated with AF can exacerbate vascular damage, accelerating the progression of cognitive impairment. Inflammatory markers are often elevated in patients with AF, contributing to a pro-thrombotic state and promoting vascular injury.

Endothelial dysfunction, a hallmark of AF, can lead to impaired blood–brain barrier integrity [77]. This dysfunction allows for the infiltration of inflammatory cells and molecules into the brain, further exacerbating neuronal injury and cognitive decline [78, 79]. The impaired endothelial function reduces the brain’s ability to regulate blood flow effectively, increasing the susceptibility to ischemic damage and contributing to the pathogenesis of VCID [74–76].

Taking together, the pathophysiological mechanisms linking AF to VCID are multifaceted, involving thromboembolism, cerebral hypoperfusion, inflammation, and endothelial dysfunction. Understanding these mechanisms is crucial for developing targeted interventions to mitigate cognitive decline in patients with AF.

## Preventive and therapeutic implications

### Early detection and prevention of VCID

Early detection of AF is critical in preventing AF-related VCID [80, 81]. Timely identification of AF allows for interventions that can mitigate the risk of stroke and cognitive decline. It is estimated that offering a rhythm check to every 5000 people aged ≥ 65 years will prevent one stroke in the first year [82]. Raising awareness through public health campaigns can play a pivotal role in educating the general population about the importance of early detection.

#### Awareness campaigns

Public health initiatives should focus on increasing awareness of AF and its association with cognitive decline. These campaigns can emphasize the importance of routine check-ups and encourage individuals to monitor their heart health actively [83–88].

Encouraging self-diagnosis techniques, such as regular pulse taking, can help individuals identify irregular heartbeats that may indicate AF [89, 90]. Educational materials and resources should be made widely available to teach people how to check their pulse accurately and recognize potential warning signs of AF.

General practicioners play a crucial role in the early detection of AF [91–94]. Training and continuous education for primary care providers can enhance their ability to recognize the signs and symptoms of AF, conduct appropriate diagnostic tests, and refer patients for specialist care when necessary.

#### Patient education

Comprehensive patient education programs are essential. These programs should inform patients about the risks associated with AF, the importance of medication adherence, and lifestyle modifications that can reduce the risk of VCID [95–99].

#### Smart devices

The use of smart devices, such as fitness trackers and smartwatches equipped with heart rate monitors, can facilitate the early detection of AF [46, 100–103]. These devices can alert users to irregular heart rhythms, prompting timely medical consultation and intervention [104].

#### Diagnosis of paroxysmal AF

Paroxysmal AF, which occurs intermittently, presents unique diagnostic challenges [105]. Traditional 24-h Holter monitoring may not capture these sporadic episodes. Longer-term monitoring using wearable devices or implantable loop recorders can improve the detection rates of paroxysmal AF, ensuring timely diagnosis and treatment. Moreover, in high-risk patients, implantable loop recorders appear to have a significantly greater rate of AF detection that external loop recorders (15% versus 5%) [106].

### Anticoagulation therapy

Anticoagulants, such as warfarin and direct oral anticoagulants (DOACs), play a crucial role in reducing stroke risk in AF patients. By preventing the formation of blood clots, these medications can mitigate cognitive decline [18, 107–113]. However, the benefits of anticoagulation must be weighed against the risk of bleeding, particularly in elderly patients. Individualized treatment plans that consider the patient’s overall health, comorbid conditions, and risk factors for bleeding are essential for optimizing outcomes.

In addition, atrial appendage closure procedures may be considered as an adjuct or alternative AF therapy to anticoagulation [114]. In particular, atrial appendage closure to reduce the risk of stroke and thromboembolism in AF may be a consideration in patients at high risk of hemorrhagic complications from anticoagulation, such as patients with cerebral amyloid angiopathy. Cerebral amyloid angiopathy, characterized by cerebrovascular deposition of amyloid β, is a common age-related small vessel pathology associated with intracerebral hemorrhage and independely associated with cognitive decline [115–118]. In its early stages, cerebral amyloid angiopathy is characterized by alteration of cerebrovascular physiology and non-hemorrhagic brain injury that then gives way to hemorrhagic brain injury in later stages [119]. The presence of higher risk cerebral amyloid angiopathy phenotypes, such as transient focal neurologic episodes, disseminated and focal cortical superficial siderosis, and lober hemorrhage with cerebral microbleeds, may inform an individualized approach to AF management that includes atrial appendage closure as a means to avoid long-term anticoagulation exposure [117].

### Rate and rhythm control

Strategies to control heart rate or rhythm in AF patients can impact cognitive outcomes [120–122]. Rate control medications aim to regulate the heart rate, while rhythm control strategies, which include medications and arrhythmia ablation procedures [123], attempt to restore and maintain normal sinus rhythm. The choice between rate and rhythm control should be based on individual patient characteristics and the potential benefits of each approach.

### Multifactorial approaches

Managing comorbid conditions that contribute to both AF and VCID, such as hypertension [124] and diabetes [125, 126], is vital. A multifactorial approach that includes lifestyle modifications is essential for reducing the risk of cognitive decline.

Adopting a heart-healthy diet, such as the Mediterranean diet, can reduce the risk of cardiovascular diseases and cognitive decline [127, 128]. Regular physical activity improves cardiovascular health and can help manage risk factors such as hypertension and diabetes [129]. Quitting smoking reduces the risk of cardiovascular diseases, stroke, and microvascular dysfunction and associated cognitive decline. Limiting alcohol intake can decrease the risk of AF and its complications.

A comprehensive approach that addresses all contributing factors is necessary for optimal patient outcomes. Collaborative care models involving cardiologists, neurologists, primary care providers, and other healthcare professionals can ensure that patients receive holistic and effective treatment plans tailored to their specific needs. Additionally, novel diagnostic tests exploring the interplay between the heart and brain may provide potential tools to identify patients at high risk for cerebral ischemia [130, 131].

## Impact of the COVID-19 pandemic on AF and cognitive decline

The COVID-19 pandemic has had a profound impact on healthcare delivery, particularly for the elderly population [132]. During the pandemic, many older adults did not receive adequate care due to several factors, including lockdowns, fear of contracting the virus, and overwhelmed healthcare systems [133, 134]. This section explores how these challenges affected the diagnosis and management of AF and subsequently contributed to cognitive decline and dementia.

### Reduced access to healthcare

During the pandemic, general practitioners and cardiologists were often unable to see patients regularly. Routine check-ups and elective procedures were postponed or canceled, leading to delays in diagnosing and managing chronic conditions like AF. Telehealth services, although beneficial, were not universally accessible or utilized by older adults, further limiting their access to necessary medical care.

### Patient reluctance to seek care

Many elderly individuals avoided seeking medical care due to fear of exposure to COVID-19. In fact, some studies suggest that reluctance to seek medical care and stringent public health measures during the pandemic may have contributed to regional decreases in patients presenting for acute ischemic stroke [132, 135]. This reluctance also likely led to a significant number of AF cases going undiagnosed for extended periods, often for years. Without regular monitoring and timely intervention, these patients were at a higher risk of developing complications, including strokes and microstrokes.

### Lax family supervision

The pandemic also led to a reduction in family supervision for many elderly individuals. Restrictions on travel and social gatherings meant that family members visited their older relatives less frequently. This lack of regular contact and oversight resulted in missed opportunities to observe changes in health status that could indicate underlying conditions such as AF. Family members often play a critical role in recognizing symptoms and encouraging medical consultations, and their reduced involvement during the pandemic further exacerbated the problem of undiagnosed AF and its complications.

### Undiagnosed strokes and microstrokes

The delay in diagnosing AF during the pandemic contributed to an increase in undiagnosed strokes and microstrokes. These cerebrovascular events often went unnoticed without the routine diagnostic measures usually in place. Over time, the accumulation of these undetected strokes led to significant brain damage and an accelerated decline in cognitive function in many patients.

### Increased cognitive decline

The combination of undiagnosed AF and subsequent strokes contributed to a significant increase in cognitive decline among the elderly during the pandemic [136–139]. Many patients who might have otherwise been diagnosed and treated early experienced more severe cognitive impairments due to the prolonged lack of medical intervention.

### Specific challenges faced

The pandemic strained healthcare resources, making it challenging for patients to access specialist care. GPs and cardiologists were often redeployed to COVID-19 care units, further reducing the availability of routine cardiovascular care. Hospitals and clinics were overwhelmed with COVID-19 cases, leading to the postponement of non-emergency procedures and consultations. This shift in focus resulted in the neglect of chronic conditions like AF. While telehealth emerged as an alternative to in-person visits, many older adults faced barriers in accessing and using these technologies. Lack of familiarity with digital tools and limited internet access contributed to reduced utilization of telehealth services.

### Long COVID and AF

COVID-19 can lead to a range of cardiovascular complications, including AF [140, 141]. Understanding the relationship between long COVID [142, 143], a condition characterized by persistent symptoms following an acute COVID-19 infection, and AF is crucial for developing effective management strategies for patients experiencing prolonged symptoms.

Long COVID is associated with a sustained inflammatory response, which can affect the heart and vascular system [144–146]. Chronic inflammation, which also include autoimune reactions [147], can damage cardiac tissues, potentially leading to arrhythmias such as AF. Additionally, the SARS-CoV-2 virus can directly infect cardiac cells, causing myocarditis and other structural changes [148–152]. These changes can create a substrate for the development of AF. Damage to small blood vessels, resulting in microvascular dysfunction, has been observed in long COVID patients [145, 152–158]. This can lead to impaired perfusion of cardiac tissues and contribute to the onset of AF.

### Vaccine side effects and AF

While COVID-19 vaccines have been crucial in controlling the pandemic and preventing severe illness, there have been reports of side effects, including potential cardiac complications such as AF [159, 160]. In rare cases, some individuals have experienced AF shortly after receiving a COVID-19 vaccine. These occurrences are relatively uncommon and typically mild, often resolving without long-term effects. The mechanisms behind vaccine-induced AF are not fully understood but may involve transient inflammatory responses. It is important for healthcare providers to monitor and manage any cardiac symptoms post-vaccination.

## Conclusion

In conclusion, atrial fibrillation is intricately linked to vascular contributions to cognitive impairment and dementia. Early detection and comprehensive management of AF are crucial to mitigating the risk of cognitive decline. Public awareness campaigns, advanced diagnostic tools, patient education, and proactive screening are essential components in identifying AF early and preventing its complications. Effective management strategies, including anticoagulation therapy, rate and rhythm control, and addressing comorbid conditions through lifestyle modifications, can significantly reduce the risk of VCID.

By addressing both the cardiac and cognitive aspects of AF, healthcare providers can significantly improve patient outcomes and quality of life. Collaborative care models and individualized treatment plans tailored to patient needs are pivotal in managing AF and preventing cognitive decline. Continued research and innovation in treatment strategies will further enhance our ability to combat the dual burden of AF and dementia, ultimately improving the health and well-being of the aging population.
